# Factors Associated With Paradoxical Masseteric Bulging After Botulinum Toxin Injection for Masseter Hypertrophy: A Retrospective Analysis

**DOI:** 10.1111/jocd.70830

**Published:** 2026-04-25

**Authors:** Yi‐dan Sun, Xiang‐wen Xu, Lin Luo, Yan‐ting Ou, Yong‐yan Cui, Dan‐dan Liu

**Affiliations:** ^1^ Department of Plastic Surgery Peking University Shenzhen Hospital Shenzhen P. R. China; ^2^ Department of Plastic Surgery Shenzhen Xinhua Hospital Shenzhen P. R. China

**Keywords:** botulinum toxin type a, masseter hypertrophy, paradoxical masseteric bulging

## Abstract

**Background:**

Botulinum neurotoxin (BoNT) injection is the preferred minimally invasive treatment for masseter hypertrophy, but paradoxical masseteric bulging (PMB) is a distressing complication. How to clinically prevent and avoid the development of PMB remains a key concern for injecting physicians.

**Methods:**

This study collected ultrasound and injection data from 22 PMB masseter muscles and 66 non‐PMB masseter muscles between September 2024 and January 2025. Univariate analysis was used to compare imaging and injection‐related parameters between the two groups, including masseter prominence, masseter thickness, deep inferior tendon (DIT) type, DIT thickness, intraoperative tactile sensation, and injected agent.

**Results:**

Univariate analysis showed significant intergroup differences in masseter prominence grade, DIT type, DIT thickness, masseter thickness, injection dosage, and intraoperative tactile sensation (all *p* < 0.05). The PMB group had greater masseter thickness (13.30 ± 0.171 mm vs. 10.32 ± 0.169 mm), thicker DIT (0.85 [0.348] mm vs. 0.60 [0.208] mm), and a higher incidence of fascial penetration sensation than the control group.

**Conclusion:**

Preoperative ultrasound assessment of masseter and DIT characteristics, combined with individualized layered injection and intraoperative tactile feedback, effectively mitigates PMB risk. Ultrasound‐guided precise supplementary injection is the preferred intervention for PMB management.

## 1 Background

The growing demand for facial contour refinement has spurred innovations in non‐surgical treatment modalities for masseter hypertrophy. Botulinum neurotoxin (BoNT) injection, characterized by its minimally invasive nature, safety profile, and predictable outcomes, has emerged as the preferred clinical modality [1]. First utilized for the treatment of masseter hypertrophy in 1994, this technique reduces masseter volume and reshapes facial contours by inhibiting acetylcholine release at the neuromuscular junction [2]. Corresponding anatomical research and the refinement of injection protocols have continued to progress. To avoid unintended drug diffusion into the risorius muscle (which may cause an unnatural smile), the conventional injection approach involves vertical needle insertion into the inferior portion of the masseter muscle for deep‐layer administration [1, 3, 4]. However, this approach has been associated with complications, including paradoxical masseteric bulging, hypersensitivity reactions, edema, ecchymosis, muscle weakness, and other facial manifestations [5, 6, 7].

Among these complications, the development of “paradoxical masseteric bulging” (PMB), colloquially referred to as “frog cheek,” has emerged as a critical factor compromising treatment outcomes. Lee et al. first documented this phenomenon in 2012 [8]. Its incidence ranges from 0.15% to 27.3% and occurs 2–4 weeks after a botulinum neurotoxin injection, with substantial variability attributed to injection techniques, anatomical heterogeneity, and assessment criteria [9, 10]. In 2016, Lee et al. introduced the concept of the “deep inferior tendon (DIT)” [11], which is believed to be implicated in PMB pathogenesis. Anatomical investigations confirmed that the DIT originates from the superficial muscular fibers of the masseter muscle and inserts inferiorly onto the mandibular margin. Acting as a physical barrier, the DIT impedes the uniform diffusion of BoNT within the superficial layer of the masseter muscle, leading to abnormal protrusion of the superficial muscle belly during contraction—thus providing the first anatomical explanation for PMB. Subsequently, during the ultrasound examination, the presence of DIT was also detected, and several scholars classified the morphology of DIT [12, 13, 14, 15]. Traditional injection techniques fail to account for this anatomical heterogeneity, contributing to the relatively high incidence of PMB. Although ultrasound‐guided injection has been shown to reduce PMB risk by addressing DIT‐related drug diffusion issues, its clinical application is limited by substantial financial and human resource requirements, precluding large‐scale implementation [15, 16, 17].

Despite the well‐established association between the DIT and PMB in current literature, critical knowledge gaps remain: first, most existing studies focus on DIT anatomical and imaging classification but lack correlations with patients' clinical presentations and PMB development, limiting the ability to guide individualized treatment; second, PMB management strategies have not been standardized. Although existing literature suggests that supplementary superficial BoNT injections may alleviate PMB symptoms, optimal injection site selection, dosage determination, and injection depth remain to be validated.

## 2 Materials and Methods

### 2.1 Patients

This case–control study enrolled 22 PMB masseter muscles that developed paradoxical masseteric bulging (PMB) within 1 month after botulinum toxin injection for masseter hypertrophy at our institution between September 2024 and January 2025, as well as 66 non‐PMB masseter muscles that underwent masseter injection during the same period without PMB occurrence. A 1:3 case–control matching was performed for the PMB and control groups on the basis of age and gender to balance baseline characteristics (Table S1). Inclusion criteria: (1) Patients aged 18–40 years with self‐reported masseter hypertrophy, an irregular mandibular contour, and who underwent BoNT injection for facial slimming at our institution. Exclusion criteria: (1) Prior BoNT facial slimming or other injection therapies within 6 months before enrollment; (2) Planned pregnancy within 6 months after injection; (3) History of autoimmune diseases, hematologic disorders, or neuromuscular diseases; (4) Known hypersensitivity to BoNT or its components; 5. Concurrent use of aminoglycoside antibiotics; 6. Active infection or inflammation at the intended injection site; (7) Missing imaging or injection‐related data. All patients underwent pre‐injection and 1 month post‐injection photography (frontal, 45° oblique, and lateral views) and had detailed injection data recorded (agent, dosage, injection sites, tactile sensation). Additionally, all patients underwent pre‐injection and 1 month post‐injection B‐ultrasound evaluation. This study was conducted in compliance with the Declaration of Helsinki guidelines, and ethical approval was obtained from the Institutional Review Board of our hospital.

### 2.2 Ultrasound Imaging

Patients underwent B‐ultrasound examinations pre‐injection and 1 month post‐injection. All ultrasound scans were performed in the Department of Ultrasonography by a single experienced operator using a Toshiba Nemio SSA‐550 ultrasound system equipped with a 7.5–9.0 MHz broadband transducer. Prior to ultrasound examination, surface landmarks were marked on each patient: commissure to earlobe (ME) line, mandibular margin, and anterior/posterior masseter margins. Ultrasound measurements were performed in the region 1 cm inferior to the ME line [18, 19]. The morphology of the deep inferior tendon (DIT) is classified into three types: compartment type, transverse type, and longitudinal type on the basis of the criteria described by Li et al. [14]. The thickness of the masseter muscle was measured at its widest point in the middle section. For DIT thickness measurement, three points were selected in this study: the thickest region of the DIT fascia, and two additional points 5 mm from this thickest site on its left and right sides. The mean value of these three points was defined as the final DIT thickness. All B‐ultrasound measurements were single‐blinded, with investigators unaware of patients' group allocations.

### 2.3 Botulinum Toxin Injection Technique

All BoNT injections were performed by a single board‐certified plastic surgeon with extensive clinical experience. Safety marking lines were defined as follows: the line connecting the earlobe and oral commissure, the inferior border of the mandible, and the anterior and posterior borders of the masseter muscle [20]. Injection dose: According to the dose–thickness relationship of the masseter muscle reported by Xie et al. [3], combined with the injector's clinical experience, an appropriate injection dose of 20–50 U per side was selected on the basis of the thickness of the patient's masseter muscle. Injection site: On the basis of the 3‐point and 5‐point injection techniques reported previously [21, 22], injections were administered 1 cm within the safety marking lines according to the size of the patient's masseter muscle and the physician's clinical experience. Injection method: In this study, the injection points of the masseter muscles all used disposable BD syringes (27G, 13 mm) and were treated by a layered injection method. The specific methods are as follows: Using a BD syringe, the needle was inserted vertically at the injection point on the masseter muscle. After fully inserting the needle until the tip reached the periosteum, 80% of the single dose was injected into the deep layer of the masseter muscle. Then the needle was withdrawn from the periosteum layer, and the remaining 20% of the single dose was given to the middle layer of the masseter muscle (superficial layer) [23]. Intraoperative tactile sensations during needle penetration were categorized: cases where fascial penetration sensation was experienced at ≥ 50% of injection sites were classified into the “Fascial Sensation Group”; the remainder were assigned to the “Ordinary Group”.

### 2.4 Statistical Analysis

Statistical analyses were conducted using R software (version 4.4) and SPSS Statistics 24.0. Sample size calculation was performed on the basis of a prior pilot study, assuming a PMB incidence of 25%, effect size of 0.8, α = 0.05, and β = 0.2, indicating that a minimum of 76 injection sites would be required to detect significant differences. Normality of continuous variables was tested using the Shapiro–Wilk test. Initial intergroup comparisons were performed: the chi‐square test was used for categorical variables, and the independent samples *t*‐test was employed for normally distributed continuous variables, and for non‐normally distributed continuous variables, non‐parametric tests (Mann–Whitney U test) were applied. Statistical significance was set at *p* < 0.05.

## 3 Result

**FIGURE 1:**
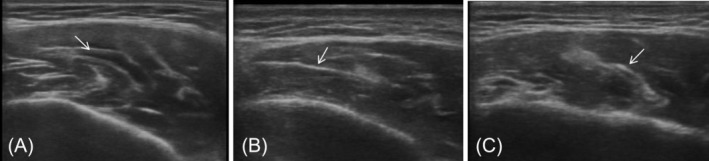
Deep inferior tendon (DIT) types: (A) Compartment type; (B) transverse type; (C) longitudinal type. Arrows indicate the DIT.

A total of 88 masseter injection sites were enrolled in this study. All patients underwent preoperative assessment of lower facial masseter prominence using the 10‐point Photonumeric Masseter Prominence Rating Scale. Grade distribution was as follows: Grade I (6/88, 6.82%), Grade II (34/88, 38.64%), Grade III (24/88, 27.27%), Grade IV (16/88, 18.18%), and Grade V (8/88, 9.01%). On the basis of the cross‐sectional morphological characteristics of the DIT within the masseter muscle, DIT was classified into three types: compartment type (31/88, 35.23%), transverse type (38/88, 43.18%), and longitudinal type (19/88, 21.59%) (Figure 1) [14].

Subsequent analysis demonstrated a significant difference in DIT type distribution between the PMB group and the control group (*p* = 0.013). At 1 cm inferior to the ME line, the mean maximum resting masseter thickness measured via ultrasound was 11.06 ± 0.213 mm. A significant difference in masseter thickness was observed between the PMB group and the control group (13.30 ± 0.171 mm vs. 10.32 ± 0.169 mm, *p* < 0.001). Additionally, the PMB group had a significantly greater average DIT thickness compared with the control group (0.85 [0.348] mm vs. 0.60 [0.208] mm, *p* < 0.001).

In terms of injection‐related factors, this study identified significant intergroup differences in injection dosage and intraoperative tactile sensation between the PMB and control groups (Table 1). Specifically, the PMB group received a significantly higher injection dosage (40.0 [4.00] U vs. 35.0 [8.00] U, *p* < 0.001) compared with the control group, a finding consistent with the clinical principle that larger masseter muscles require a relatively higher BoNT dosage to achieve optimal outcomes. Regarding intraoperative tactile sensation, 72.7% of cases in the PMB group reported fascial penetration sensation during injection, significantly higher than the 15.1% in the control group (χ^2^ = 26.28, *p* < 0.001). This fascial sensation is believed to indirectly reflect the wide distribution and increased toughness of the DIT at the injection plane, which may impede uniform BoNT diffusion and thereby elevate PMB risk. Univariate analysis revealed significant differences between the PMB and non‐PMB groups in masseter prominence grade, masseter thickness, and intraoperative tactile sensation.

**TABLE 1:** Univariate Analysis of Clinical and Imaging Factors Associated with Paradoxical Masseteric Bulging (PMB: 22 masseter muscles; Control: 66 masseter muscles).

Variables	PMB (*N* = 22)	Control (*N* = 66)	Test statistic	*p*
10‐point Photonumeric Masseter Prominence Grade, *n* (%)				
I	0 (0.0)	6 (9.1)	χ^2^ = 22.961	0.000***
II	2 (9.1)	32 (48.5)		
III	6 (27.3)	18 (27.3)		
IV	9 (40.9)	7 (10.6)		
V	5 (22.7)	3 (4.5)		
DIT classification, *n* (%)				
Compartment type	9 (40.9)	22 (33.3)	χ^2^ = 8.321	0.016**
Transverse type	13 (59.1)	25 (37.9)		
Longitudinal type	0 (0.0)	19 (28.8)		
Thickness of the masseter muscle (mm), Mean ± SD	13.30 ± 0.171	10.32 ± 0.169	*t* = 7.174	0.000***
DIT average thickness (mm), Median (IQR)	0.85 (0.348)	0.60 (0.208)	Z = −3.946	0.000***
Injected agent, *n* (%)				
Botox	17 (77.3)	53 (80.3)	χ^2^ = 0.093	0.760
Lantox	5 (22.7)	13 (19.7)		
Injection dosage (U), Median (IQR)	40.0 (4.00)	35.0 (8.00)	Z = −3.946	0.000***
Intraoperative tactile sensation, *n* (%)				
Fascial Sensation	16 (72.7)	10 (15.1)	χ^2^ = 26.276	0.000***
Ordinary group	6 (27.3)	56 (84.8)		

*Note:* *DIT: deep inferior tendon; IQR: interquartile range. Data are presented as *n* (%), Mean ± SD or Median (IQR) as appropriate. χ^2^: chi‐square test; t: independent samples *t*‐test; Z: Mann–Whitney U test. *p < 0.05, **p < 0.01, ***p < 0.001.

## 4 Treatment of PMB

**FIGURE 2:**
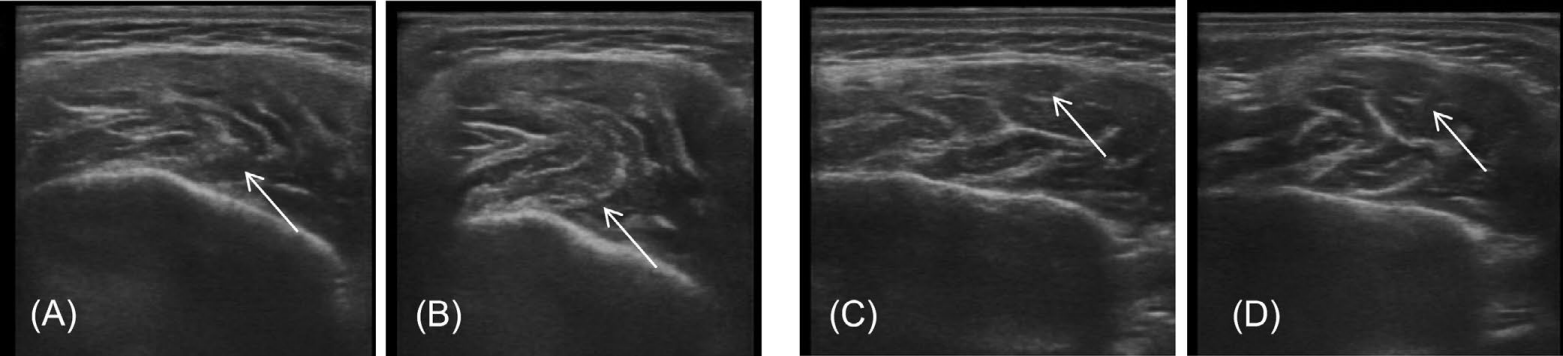
Paradoxical masseteric bulging (PMB) types: (A, B) PMB caused by deep masseter muscle contraction; (A) resting state; (B) clenched contraction state; (C, D) PMB induced by superficial masseter muscle contraction; (C) resting state; (D) clenched contraction state. The arrow indicates the location of the abnormal protrusion.

A total of 22 masseter muscles with PMB were enrolled in this study, with a mean onset time of 5.41 ± 1.74 days postoperatively. Notably, 10 patients with PMB showed no improvement 1 month after the initial injection. Subsequent postoperative ultrasound examinations revealed that 45.5% of PMB patients exhibited deep masseter muscle contraction, whereas 54.5% demonstrated superficial masseter muscle contraction (Figure 2). On the basis of ultrasound findings, supplementary BoNT injections (5–15 U) were administered either deeply or superficially, and PMB resolved within 1 week of the supplementary injection.

## 5 Case

**FIGURE 3:**
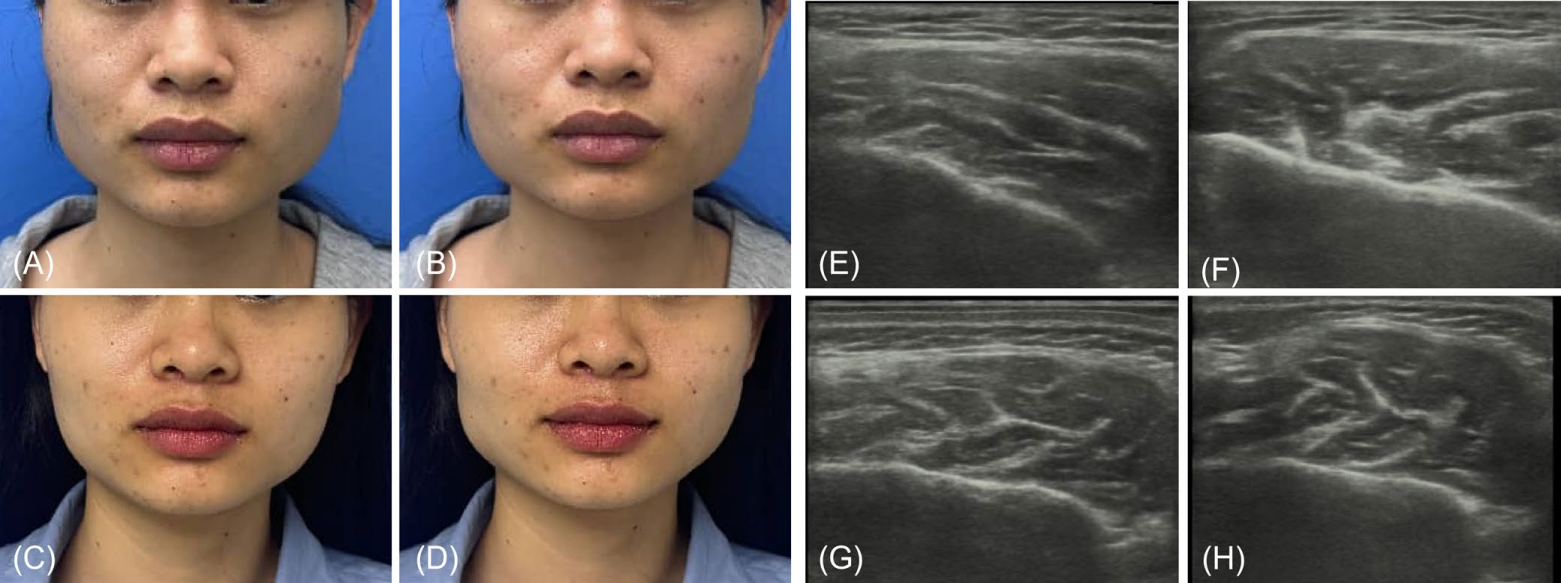
Case: A 27‐year‐old female. Preoperative photographs: (A) Resting state; (B) clenched contraction state. One‐month postoperative photographs: (C) Resting state; (D) clenched contraction state, showing abnormal bulging of the right masseter muscle. Pre‐injection B‐ultrasound images: (E) Resting state; (F) clenched contraction state. One‐month post‐injection B‐ultrasound images: (G) Resting state; (H) clenched contraction state, demonstrating abnormal contraction of the superficial masseter muscle.

A 27‐year‐old female presented with Grade V masseter prominence and a multi‐bellied masseter muscle. Ultrasound examination revealed a compartment‐type deep inferior tendon (DIT) on the left side and a transverse‐type DIT on the right side, with the masseter muscle measuring 14.8 mm on the left and 15.2 mm on the right. She received 40 U of Botox (8 U injected superficially) on the left side and 42 U of Botox (8 U injected superficially) on the right side. Intraoperatively, no fascial penetration sensation was noted on the left side (classified as the “Ordinary Group”), whereas fascial penetration sensation was observed at multiple injection sites on the right side (classified as the “Fascial Sensation Group”). Right‐sided paradoxical masseteric bulging (PMB) developed 3 days postoperatively. A follow‐up ultrasound examination confirmed abnormal contraction of the superficial right masseter muscle (Figure 3). A supplementary injection of 10 U botulinum neurotoxin (BoNT) was administered, resulting in excellent clinical outcomes.

## 6 Discussion

Botulinum toxin type A (BoNT‐A) injection is a widely utilized, convenient modality for facial volume reduction and contour refinement. However, multiple factors can influence the final aesthetic outcome and may induce adverse events that compromise patients' quality of life. Specifically, paradoxical masseteric bulging (PMB), colloquially termed “frog cheek,” is a distressing adverse event that impairs postoperative facial aesthetics. The presence of the DIT leads to uneven BoNT diffusion between the superficial and deep masseter bellies, which prior studies have identified as a primary pathogenic factor for PMB following masseter injection [11]. Multiple studies have verified the existence of the DIT via cadaveric dissections and ultrasound examinations, and have delineated DIT morphological classifications [13, 15]. Although numerous studies have reported the occurrence of PMB and identified potential anatomical factors, its underlying mechanism remains unclear, and it is almost impossible to establish its internal location through clinical assessment [24]. How to prevent the occurrence of PMB remains a topic of ongoing discussion among injecting physicians. Previous studies have demonstrated that ultrasound‐guided injection can effectively reduce the incidence of PMB [16]. However, this approach is clinically uneconomical, as it requires substantial human and material resources, making its implementation relatively challenging [17, 25]. Therefore, identifying PMB‐related risk factors, selecting appropriate injection techniques, and counseling patients regarding potential risks are more clinically relevant in routine practice. Thus, the present study aimed to investigate potential factors associated with PMB, including masseter thickness, DIT classification, intraoperative tactile sensation, and BoNT agent, to comprehensively elucidate the determinants of PMB development and offer evidence‐based guidance for clinical practice.

This case–control study enrolled 22 masseter muscles with PMB and 66 non‐PMB masseter muscles. Consistent with prior anatomical studies, the DIT was identified in all 88 masseter injection sites in the present study. DIT was classified into three subtypes: compartment type (31/88, 35.23%), transverse type (38/88, 43.18%), and longitudinal type (19/88, 21.59%) [14]. Notably, PMB predominantly developed in masseter muscles with compartment‐type or transverse‐type DIT, whereas it was rarely observed in those with longitudinal‐type DIT (χ^2^ = 8.321, *p* = 0.016). This observation may be attributed to the fact that compartment‐type and transverse‐type DIT are perpendicular to the injection direction, thereby increasing the likelihood of impeding BoNT diffusion. In contrast, most of the longitudinal‐type DIT runs parallel to the injection path, having less impact on drug distribution. Furthermore, the PMB group exhibited a significantly greater average DIT thickness compared with the control group (0.85 [0.348] mm vs. 0.60 [0.208] mm, *p* < 0.001). This finding suggests that increased DIT thickness may further hinder BoNT diffusion, exacerbating the risk of localized muscle under‐innervation and subsequent PMB. The mean maximum resting masseter thickness measured via ultrasound was 11.06 ± 0.213 mm, which is consistent with the findings reported by Wang et al. [12]. A significant difference in masseter thickness was noted between the PMB group and the non‐PMB group (13.30 ± 0.171 mm vs. 10.32 ± 0.169 mm, *p* < 0.001). This finding may be attributable to the effect of masseter thickness on BoNT diffusion. Prior studies have suggested that PMB may arise from uneven BoNT diffusion, which subsequently induces asymmetric muscle paralysis [5, 7, 8]. However, research into the internal anatomical characteristics of the masseter muscle and the pathogenesis of PMB remains limited to date. In particular, the potential correlation between masseter muscle thickness and the morphological subtypes as well as the thickness of the DIT remains unclear and thus merits further investigation. These knowledge gaps further underscore the multifactorial complexity underlying PMB pathogenesis. These findings also suggest to clinicians that a detailed and comprehensive pre‐injection evaluation is beneficial.

Beyond the ultrasonographic analysis of masseter anatomical morphology, the present study further explored injection‐related factors. Significant differences between the two groups were observed in injection dosage and intraoperative tactile sensation (dosage: 40.0 [4.00] U vs. 35.0 [8.00] U, *p* < 0.001; intraoperative tactile sensation: χ^2^ = 26.276, *p* < 0.001). Clinically, larger masseter muscles typically require a relatively higher BoNT dosage to achieve optimal therapeutic effects. Insufficient BoNT dosage may also result in incomplete neuromuscular blockade of the masseter muscle, leaving some muscle bundles with persistent strong contraction and consequent abnormal bulging. No significant difference in the incidence of paradoxical masseteric bulging (PMB) was observed between different botulinum neurotoxin (BoNT) brands (Lantox and Botox) in this study. Previous studies have demonstrated that Lantox and Botox exhibit comparable efficacy and safety profiles, with no significant differences in their biological activity [26, 27]. In this study, no significant between‐group differences were observed in the incidence of PMB, which is consistent with prior findings. Nevertheless, this study is limited by its relatively small sample size and short follow‐up duration. Further investigations with larger cohorts are still warranted to clarify potential differences between the two brands.

Meanwhile, intraoperative tactile sensation can indirectly reflect the distribution scope and compactness of the DIT within the injection plane of the masseter muscle. The experience of fascial penetration sensation at multiple injection sites suggests that the DIT is widely distributed within the masseter belly and exhibits increased compactness, which may hinder BoNT diffusion. If fascial penetration sensation is encountered at multiple injection sites during administration, it is recommended to increase the number of injection sites, adopt layered or retrograde injection techniques, and reduce the single‐injection dosage to avoid drug accumulation.

Subsequent analysis of patients with PMB, via ultrasound examinations performed 1 month postoperatively, revealed two distinct manifestations: abnormal prominence of the superficial masseter muscle in some patients and contraction of the deep muscle layer in others. We hypothesize that this discrepancy may stem from inadequate BoNT dosage in the deep masseter layer after layered injection. This manifestation is slightly inconsistent with previous studies, supplementing the clinical phenotype spectrum of PMB and providing partial references for clinical treatment. In addition to guiding clinical injections, these findings further assist physicians in identifying PMB risk during pre‐injection consultations and explaining the expected risk of PMB to patients. For patients who develop PMB, this study recommends ultrasound‐guided, precise layered supplementary injection with a low dosage as the preferred intervention.

However, this study is a single‐center retrospective study. Additionally, the time and financial costs associated with B‐ultrasound examinations resulted in a relatively limited number of patients with complete imaging data and valid follow‐up consent, leading to a small sample size (44 patients, 88 masseter muscles). A small sample size may increase the risk of selection bias and thus compromise statistical power. Furthermore, only univariate analysis was performed in this study, precluding the identification of independent risk factors for PMB. To minimize such biases, we rigorously screened patients according to predefined inclusion and exclusion criteria and conducted case–control matching to reduce baseline characteristic imbalances. In subsequent research, we will further expand the sample size and perform multivariate analysis to identify the independent risk factors for PMB.

## 7 Conclusion

This study investigated the factors and mechanisms underlying paradoxical masseteric bulging (PMB) following botulinum toxin type A (BoNT‐A) injection for masseter hypertrophy, aiming to provide clinical guidance for reducing PMB incidence. Clinically, preoperative assessment of relevant factors (e.g., masseter muscle prominence, DIT thickness, DIT morphological subtype, and fascial sensation), intraoperative administration of an appropriate dosage, and the use of layered injection are recommended to mitigate the risk of PMB.

## Author Contributions

Methodology design and implementation: D‐d.L. and Y‐y.C., along with Y‐d.S., conducted the research data curation and wrote the original manuscript. X‐w.X. and L.L. analyzed the results. All authors reviewed the manuscript.

## Funding

This research was funded by the National Nature Science Foundation of China (82302810), the Natural Science Foundation from Shenzhen Science and Technology Innovation Commission (JCYJ20220531094004010 and JCYJ20210324105412033), the Shenzhen Key Medical Discipline Construction Fund (SZXK026), the Peking University Shenzhen Hospital Research Project Fund (LCYJ2020008 and LCYJ2022015), and the Guangdong Medical Science and Technology Research Foundation (A2023237).

## Ethics Statement

All procedures involving human participants were conducted in accordance with the 1964 Declaration of Helsinki and its subsequent amendments. This study was approved by the Peking University Shenzhen Hospital Institutional Ethics Committee (Approval No.: 2026[017]) with a waiver of written informed consent; verbal informed consent was obtained from all participants for the use of identifiable patient photographs and other personally identifiable materials in this research.

## Conflicts of Interest

The authors declare no conflicts of interest.

## Supporting information


**Table S1:** Baseline characteristics of the study population (22 PMB masseter muscles vs. 66 non‐PMB masseter muscles).

## Data Availability

The data that support the findings of this study are available on request from the corresponding author. The data are not publicly available due to privacy or ethical restrictions.
