# Supplementary material for: Factors Associated With Paradoxical Masseteric Bulging After Botulinum Toxin Injection for Masseter Hypertrophy: A Retrospective Analysis
Source: J Cosmet Dermatol. 2026 Apr 25;25(4):e70830. doi: 10.1111/jocd.70830 (PMC13109811; doi:10.1111/jocd.70830)
Supplement: Supplementary file 1 — Table S1: Baseline characteristics of the study population (22 PMB masseter muscles vs. 66 non‐PMB masseter muscles). [file JOCD-25-e70830-s001.docx]

Table S1. Baseline Characteristics of the Study Population (22 PMB masseter muscles vs. 66 non-PMB masseter muscles)

| **Variables** | **PMB**  **(N=22)** | **Control**  **(N=66)** | **Test statistic** | **P-value** |
| --- | --- | --- | --- | --- |
| **Gender, n (%)** |  | | | |
| Male | 4 (18.2) | 8 (12.1) | χ²=0.515 | 0.473 |
| Female | 18 (81.8) | 58 (87.9) |  |  |
| **Age (yr), Mean ± SD** | 26.68 ± 5.027 | 27.47 ± 4.247 | t=-0.719 | 0.474 |

Data are presented as n (%) or Mean ± SD as appropriate. χ²: chi-square test; t: independent samples t-test. No significant difference was observed between the two groups (all P>0.05).
